# Supplementary material for: Lactate Activates Germline and Cleavage Embryo Genes in Mouse Embryonic Stem Cells
Source: Cells. 2022 Feb 4;11(3):548. doi: 10.3390/cells11030548 (PMC8833948; doi:10.3390/cells11030548)
Supplement: Supplementary file 1 [file cells-11-00548-s001.zip › cells-1572826-supplementary.pdf]

cells

MDPI

Article

Lactate Activates Germline and Cleavage Embryo Genes in Mouse Embryonic Stem Cells

Qing Tian and Li-quan Zhou *

Institute of Reproductive Health, Tongji Medical College, Huazhong University of Science and Technology, Wuhan 430030, China; d202081624@hust.edu.cn
* Correspondence: zhouliquan@ hust.edu.cn

Supplementary Materials

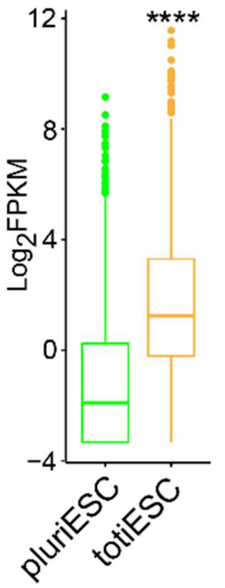

Figure S1. Global expression comparison of upregulated genes between MERVL$^+$Zscan4$^+$ ESCs and MERVL$^-$Zscan4$^-$ ESCs (GSE85776). Wilcoxon rank sum test was used to calculate p value. **** $p < 0.0001$.

Table S1. Primer sequences

Oct4-F	CTCCCTACAGCAGATCACTCA
Oct4-R	TAGCCTCATACTCTTCTCGTTG
Nanog-F	TCGCCCTTCCTCTGAAGAC
Nanog-R	TGCTTCTGAAACCTGTCCTTGA
Klf4-F	GTGCCCCGACTAACCGTTG
Klf4-R	GTCGTTGAACTCCTCGGTCT
Sox2-F	GCGGAGTGGAAACTTTTGTCC
Sox2-R	CGGGAAGCGTGTACTTATCCTT
Dazl-F	ATGTCTGCCACAACTTCTGAG
Dazl -R	CTGATTTCGGTTTCATCCATCCT
Ddx4-F	GCTTCATCAGATATTGGCGAGT
Ddx4-R	GCTTGGAAAACCCTCTGCTT
Mael-F	CCTCCCTTGTGAAATTGGCTG
Mael -R	AATGGAATCGAAATCCTCGTGG
Taf7l-F	AGCCCCCGTTATTCCTGAAG
Taf7l -R	CCTCGGGTGTCACAAGTGT
Mei4-F	CACTGGCGTTGGCGATTATC
Mei4-R	TGATTTCCATGTGGACTCCTTCT
Dmrt1-F	GACCCCGCCTACTACAGCA
Dmrt1-R	GTCTGAGCAGGCACGTAAGG
Zscan4-F	GAGATTCATGGAGAGTCTGACTGATGAGTG
Zscan4-R	GCTGTTGTTTCAAAAGCTTGATGACTTC
Zfp352-F	ACCACCTCAAAGAACACCAG
Zfp352-R	ACAAGGGACAAGCGTAGAAC

Tcstv3-F	AGAAAGGGCTGGAACTTGTGACCT
Tcstv3-R	AAAGCTCTTTGAAGCCATGCCCAG
Zfp560-F	AGAACTACCAGAACCTGGCTAC
Zfp560-R	CGCTGAAACATTGTCCCTCT
Sp110-F	AAGGATCCAGGAACCCCTTA
Sp110-R	GCATAGGCGATGTTCACCTT
Dux-F	GCTGTAGCGAGGTGAGTCGA
Dux -R	TGCCTCAAAGAGGTCCATCA
Act-F	CCAGTTGGTAACAATGCCATGT
Act -R	CCAGTTGGTAACAATGCCATGT

Table S2. GO-Up regulated genes.

GO:0003333	amino acid transmembrane transport
GO:0042138	meiotic DNA double-strand break formation
GO:0034976	response to endoplasmic reticulum stress
GO:1901605	alpha-amino acid metabolic process
GO:0043046	DNA methylation involved in gamete generation
GO:0046653	tetrahydrofolate metabolic process
GO:0045475	locomotor rhythm
GO:0001944	vasculature development
GO:0042594	response to starvation
GO:0070373	negative regulation of ERK1 and ERK2 cascade

Table S3. GO-Down regulated genes.

GO:0009615	response to virus
GO:0061762	CAMKK-AMPK signaling cascade
GO:0051960	regulation of nervous system development
GO:0003407	neural retina development
GO:0045104	intermediate filament cytoskeleton organization
GO:0048568	embryonic organ development
GO:0010665	regulation of cardiac muscle cell apoptotic process
GO:0071353	cellular response to interleukin-4
GO:0050808	synapse organization
GO:0021766	hippocampus development
